# 

**Published:** 1917-05

**Authors:** 


CONTENTS.

ORIGINAL COMMUNICATIONS .......... 33

SELECTIONS AND ABSTRACTS .......... 37

BOOK REVIEWS .......... 60

